# Identification of characteristic genes and construction of regulatory network in gallbladder carcinoma

**DOI:** 10.1186/s12920-023-01663-z

**Published:** 2023-10-11

**Authors:** Hanrui Shao, Jiahai Zhu, Ya Zhu, Lixin Liu, Songling Zhao, Qiang Kang, Yunxia Liu, Hao Zou

**Affiliations:** 1 Department of Hepatopancreatobiliary Surgery, The Second Affiliated Hospital of Kunming Medical University, 374 Dianmian Avenue, Wu Hua District, Kunming, 650106 Yunnan P.R. China; 2 Experiment Teaching Center, Basic Medical School, Kunming Medical University, 1168 West Chunrong Road, Kunming, 650500 P.R. China

**Keywords:** Gallbladder carcinoma, ceRNA network, Hsa-miR-4770, Machine learning, Bioinformatics analysis

## Abstract

**Background:**

Gallbladder carcinoma (GBC) is a highly malignant tumor with a poor overall prognosis. This study aimed to identify the characteristic microRNAs (miRNAs) of GBC and the competing endogenous RNA (ceRNA) regulatory mechanisms.

**Methods:**

The microarray data of GBC tissue samples and normal gallbladder (NGB) tissue samples from the Gene Expression Omnibus (GEO) database was downloaded. GBC-related differentially expressed miRNAs (DE-miRNAs) were identified by inter-group differential expression analysis and weighted gene co-expression network analysis (WGCNA). Machine learning algorithms were used to screen the characteristic miRNA based on the intersect between least absolute shrinkage and selection operator (LASSO) and Support vector machine-recursive feature elimination (SVM-RFE). Based on the differential expression analysis of GEO database, the ceRNA network of characteristic miRNA was predicted and constructed. The biological functions of the ceRNA network were revealed by carrying out the gene enrichment analysis was implemented. We further screened the key genes of ceRNA network and constructed a protein-protein interaction (PPI) network, and predicted and generated the transcription factors (TFs) network of signature miRNAs. The expression of characteristic miRNA in clinical samples was verified by quantitative real-time polymerase chain reaction (qRT-PCR).

**Results:**

A total of 131 GBC-related DE-miRNAs were obtained. The hsa-miR-4770 was defined as characteristic miRNA for GBC. The ceRNA network containing 211 mRNAs, one miRNA, two lncRNAs, and 48 circRNAs was created. Gene enrichment analysis suggested that the downstream genes were mainly involved in actin filament organization, cell-substrate adhesion, cell-matrix adhesion, reactive oxygen species metabolic process, glutamine metabolic process and extracellular matrix (ECM)-receptor interaction pathway. 10 key genes in the network were found to be most correlated with disease, and involved in cell cycle-related processes, p53, and extrinsic apoptotic signaling pathways. The qRT-PCR result demonstrated that hsa-miR-4770 is down-regulated in GBC, and the expression trend is consistent with the public database.

**Conclusions:**

We identified hsa-miR-4770 as the characteristic miRNA for GBC. The ceRNA network of hsa-miR-4770 may play key roles in GBC. This study provided some basis for potential pathogenesis of GBC.

**Supplementary Information:**

The online version contains supplementary material available at 10.1186/s12920-023-01663-z.

## Introduction

 As one of the common digestive tract tumors, Gallbladder carcinoma (GBC) has a high degree of malignancy, unsatisfactory survival, and prognosis [1]. According to GLOBOCAN 2020, GBC accounts for 0.9% of all cancers diagnosed but 1.7% of all cancer-related deaths [2]. Because of the absence of specific symptoms in the early stages of GBC, the vast majority of patients are diagnosed at advanced stages, and the five-year survival rate is only 10% [3]. More and more studies are coming to explore the treatment modalities for GBC. The surgical resection is only potentially curative therapy for GBC. Unfortunately, most patients with this type of cancer have unresectable disease, only 10–30% of patients can be considered for surgery on presentation [3]. Therefore, further identifying diagnostic biomarkers and potential therapeutic targets for GBC, and achieving early diagnosis and treatment to promote survival rate are the urgent task and the goal we are eager to achieve currently.

Noncoding RNAs (ncRNAs), a class of transcripts without protein-coding potential, produce noncoding transcripts that regulate gene expression and protein function [4]. The ncRNAs are typically classified into two groups according to their size: small ncRNAs of less than 200 nucleotides (nt), which include microRNAs (miRNAs), circular RNAs (circRNAs), Piwi-interacting RNAs and small nucleolar RNAs, and long noncoding RNAs (lncRNAs, >200 nt) [5]. miRNAs are typically 22 nt long and evolutionarily conserved single-stranded RNA molecules, negatively regulating the expression of protein‐coding genes [6]. There have been widely reported that miRNAs played crucial roles in development, metastasis, epithelial-mesenchymal transition, and prognosis of GBC [7]. Yi-Jun Shu et al. reported that overexpression of miR-29c-5p effectively inhibited cancer cell growth, DNA replication and colony formation [8]. Jia-Nan Chen et al. also showed that miR-139-5p significantly inhibited GBC proliferation, invasion, and glucose metabolism by targeting Pyruvate kinase isozyme type M2 (PKM2) [9]. Competing endogenous RNAs (ceRNAs) are transcripts that cross-regulate each other by competing for shared miRNAs [10]. In the ceRNA network, ncRNAs with miRNA response elements (MREs) competitively bind miRNAs to affects miRNA-mRNA binding, thereby regulating the expression of RNAs with the same MREs [11].

In this study, we employed three transcriptomic datasets associated with GBC as GSE104165, GSE76633 and GSE100363, respectively, and identified the characteristic miRNA of GBC by bioinformatic analysis. Next, based on the prediction of target molecules for key miRNAs, we constructed a potential ceRNA network for GBC. Further, we established a transcription factors (TFs) network of characterized miRNAs. Finally, quantitative real-time polymerase chain reaction (qRT-PCR) was used to detect the differences in the expression of the characteristic miRNA between the GBC samples and control samples. This study provided a basis for understanding the potential ceRNA-related pathogenesis of GBC and identify early diagnostic biomarkers and potential therapeutic targets for GBC.

## Methods

**Fig. 1:**
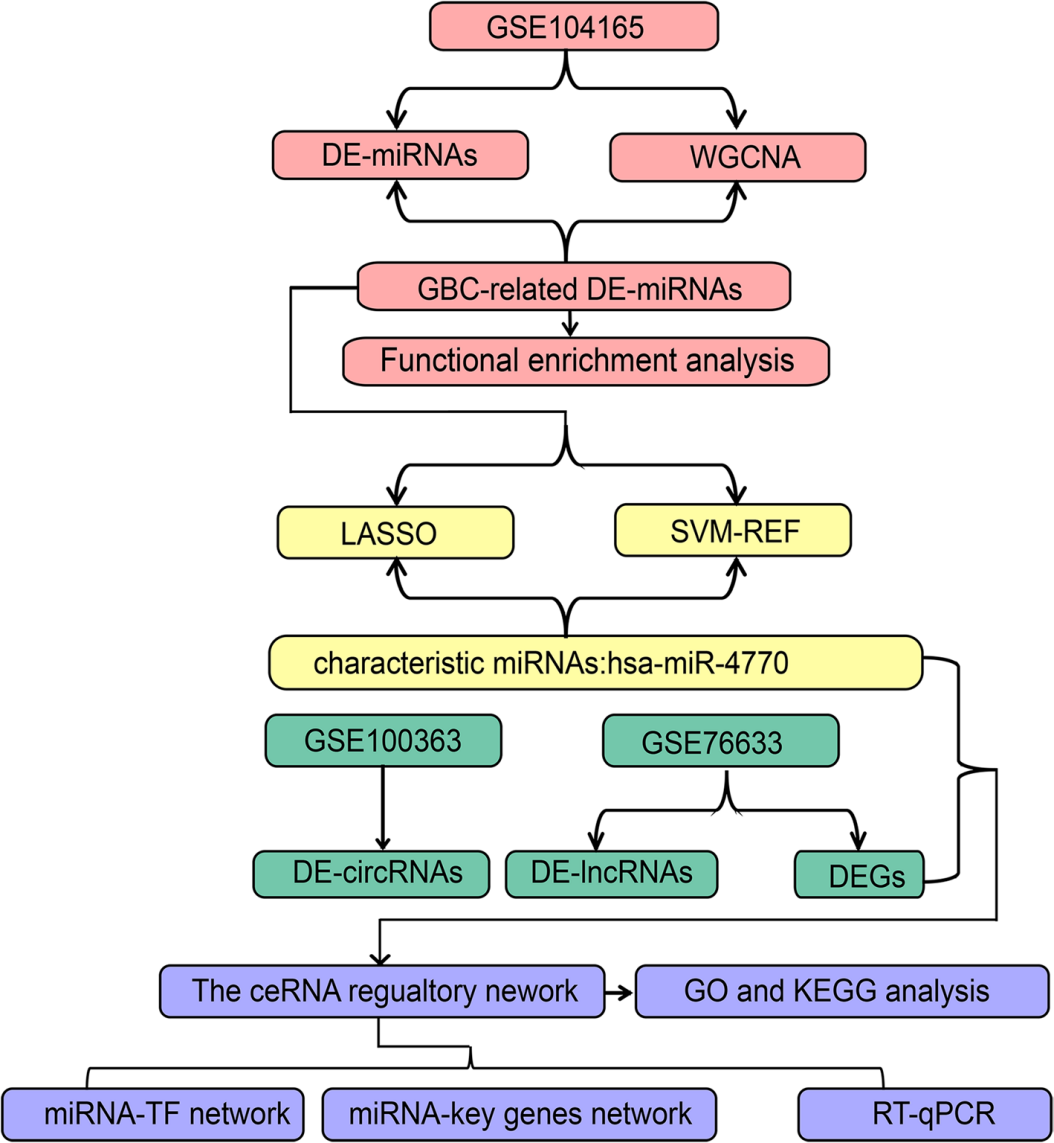
The workflow chart in this study. GBC, Gallbladder carcinoma; WGCNA, weighted gene correlation network analysis; DE, differentially expressed; LASSO: least absolute shrinkage and selection operator; SVM-RFE: Support vector machine-recursive feature elimination; DEGs, differentially expressed genes; GO: Gene Ontology; KEGG, Kyoto Encyclopaedia of Gene and Genome; TFs: Transcription factors; qRT-PCR: quantitative real-time polymerase chain reaction

The work flow of this study is shown in Fig. 1.

### Data source

Three GBC microarray datasets (GSE104165, GSE76633 and GSE100363) were screened in the Gene Expression Omnibus (GEO) database (https://www.ncbi.nlm.nih.gov/geo/). GSE104165 contains the miRNA expression profiles of eight normal gallbladder (NGB) tissue samples and 40 GBC tissue samples. GSE76633 was comprised of the mRNA and lncRNA expression profiles of nine pairs of GBC tissue samples and paired NGB tissue samples. The circRNA expression profiles of four GBC tissue samples and four matched NGB tissue samples were included in GSE100363. The summary of this dataset is shown in Table 1.

**Table 1:** Details of GBC microarray datasets profiles that we analyzed

GEO accession	Platform	Year	Country	Normal (n)	Tumor (n)
GSE104165	GPL18402	2017	Germany	40	8
GSE76633	GPL18180	2016	China	9	9
GSE100363	GPL20795	2019	USA	4	4

### Identification of differentially expressed mRNA (DE-mRNAs), miRNAs (DE-miRNAs), lncRNAs (DE-lncRNAs) and circRNAs (DE-circRNAs)

The DE-mRNAs, DE-miRNAs, DE-lncRNAs, and DE-circRNAs between GBC and NGB samples were identified by “limma” R package [12]. The cut-off criterion was |log2 Fold Change (FC)|> and *P*-value < 0.05. The corresponding volcano maps and heatmaps were created using the “ggplot2” and “pheatmap” R package respectively.

### Weighted gene co-expression network analysis (WGCNA)

The expression data of GSE104165 dataset was analyzed using the “WGCNA” R package [13] to construct the co-expression network which utilized the tumor and survival as clinical traits. The “good Samples Genes” function was used to perform sample clustering to identify and remove outliers. For making the co-expression network contented the distribution of scale-free network, a soft-thresholding power was computed with the “pick Soft Threshold” function. The dynamic tree cutting method was used to identify different modules with the minimum number of genes in each module was 30. Subsequently, a merging threshold of 0.45 was set to merge similar modules. The correlation between these modules and clinical traits (tumor and survival) was further analyzed. Finally, the module with the highest Pearson correlation coefficient with clinical traits (tumor and survival) was chosen as key module, in which miRNAs with |MM|>0.8 and |GS|>0.2 were identified as key module genes, namely hub genes [14]. Next, the GBC-related DE-miRNAs were identified by overlapping the hub genes and DE-miRNAs, the function annotation for these overlapped miRNAs was conducted using miEAA (https://ccb-compute2.cs.uni-saarland.de/mieaa2/) [15].

### Screening for characteristic miRNAs by machine learning

The “glmnet” R package was utilized to screen for characteristic miRNAs using the least absolute shrinkage and selection operator (LASSO) regression analysis [16]. The area under the receiver operating characteristic curve (ROC) was used to assess the diagnostic sensitivity and specificity of the LASSO model. Support vector machine-recursive feature elimination (SVM-RFE) is a machine learning method in terms of support vector machine algorithms, which can effectively derive a subset of informative genes and make the classification more reliable [17]. The characteristic miRNAs were filtered out by the “e1071” R package with the ten-fold cross-validation method. Eventually, characteristic miRNAs, namely, potential biomarkers, were determined by overlapping the miRNAs identified by LASSO and SVM-RFE. The classification performance of characteristic miRNAs between GBC and NGB samples was evaluated using the area under the ROC curve, which was drawn by “pROC” R package [18].

### Construction of ceRNA nework

We performed target genes (mRNAs), upstream circRNAs, and upstream lncRNAs prediction for the characteristic miRNAs using the Starbasedatabase (http://starbase.sysu.edu.cn/). The predicted mRNAs, circRNAs, lncRNAs, and the corresponding DE-mRNAs, DE-circRNAs, DE-lncRNAs were intersected according to the ceRNA regulatory mechanism. Finally, the lncRNA (circRNA)-miRNA-mRNA network was created by “Cytoscape” software [19].

### Functional enrichment analysis

Gene Ontology (GO) and KEGG enrichment analysis was implemented by “clusterProfiler” R package [20]. The threshold for significance was *P*-value<0.05. GO enrichment analysis mainly described the biological processes (BP), cellular components (CC), and molecular functions (MF) correlated with genes.

### Screening for key genes by varElect

We applied varElect tothe genes in the ceRNA network for disease linkage using the keywords “gallbladder carcinoma” and “cancer”. The varElect collected information based on relevant literature reports and databases for disease linkage detection [21, 22]. The higher the score value, the greater the probability of disease linkage.

#### Interaction network analysis by GeneMANIA

GeneMANIA (http://www.genemania.org) is a database focused on gene function and related genes, including co-expression, physical interactions, genetic interactions, co-localization, and pathways. We accessed GeneMANIA to search for functions and interactions associated with key genes, and enriched the protein-protein interaction (PPI) network.

### Construction of the miRNA-Transcription factors (TFs) network by TransmiR

In order to investigate the regulatory mechanism of characteristic miRNAs, we utilized TransmiR (http://www.cuilab.cn/transmir) to predict the characteristic miRNAs associated TFs and generate the miRNA-TFs network [23].

### Patients and specimens

The study was confirmed by the China Ethics Committee of Registering Clinical Trials (Ethical review number: Chi ECRCT20190065). After obtaining informed consent from all participants, 20 tissue specimens were selected from 10 patients with GBC, including 10 tumor tissues and 10 paired adjacent normal gallbladder tissues. The selected GBC patients underwent surgery at the Second Affiliated Hospital of Kunming Medical University from June 2020 to June 2021, and the pathological diagnosis was finally diagnosed as GBC.

### RNA extraction and quantitative real-time polymerase chain reaction (qRT-PCR)

Total RNA from fore-mentioned 10 NGB tissue samples and 10 GBC tissue samples was isolated using the TRIzol Reagent following the manufacturer’s instructions (Ambion, USA). Next, total RNA was reverse transcribed into cDNA utilizing the SweScript-First-strand-cDNA-synthesis-kit (Servicebio, China), according to the manufacturers’ protocol. qRT-PCR was subsequently performed using the 2×Universal Blue SYBR Green qPCR Master Mix (Servicebio, China). The following thermocycling conditions were used for qPCR: one cycle at 95 °C for 60 s (initial denaturation), followed by 40 cycles of 20 s at 95 °C (denaturation), 20 s at 55 °C (annealing), and 30 s at 72 °C (extension). The relative expression level was normalized to the endogenous control U6 and calculated using the 2 − ΔΔCq method [24]. The primers of the target genes were: hsa-miR-4770-F: TGAGATGACACTGTAGCT, hsa-miR-4770-R: GTCGTATCCAGTGCAGGGT; U6-F: CTCGCTTCGGCAGCACA, U6-R: AACGCTTCACGAATTTGCGT.

### Statistical analysis

All statistical analyses were performed using R software (version 4.3.1) and its appropriate packages One-way ANOVA analysis was used to compare data between GBC and NGB groups. ROC curve was generated to evaluate the sensitivity and specificity in distinguishing GBC and NGB samples. If not specified above, a *P*-value less than 0.05 was considered statistically significant.

## Results

### Identification of GBC-related DE-miRNAs

**Fig. 2:**
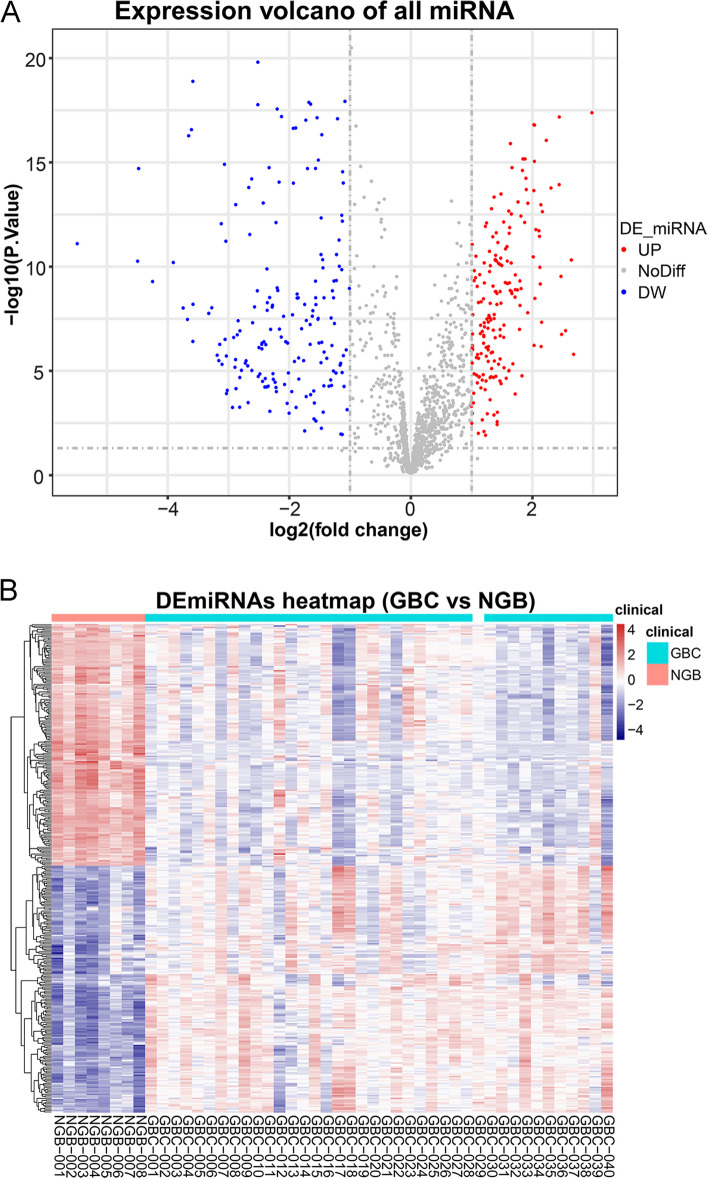
Identification of differentially expressed miRNAs between GBC and NGB samples. **A** The volcano plot showed that a total of 182 upregulated and 172 downregulated DE-miRNAs were screened out (|log_2_ Fold Change (FC)|>1.0 and *P*-value<0.05). Each dot represents a miRNA, with red, blue, and gray representing upregulated differential miRNAs, downregulated differential miRNAs, and nonsignificant differential miRNAs, respectively. **B** The heatmap exhibited the expression levels of 354 DE-miRNAs of each sample in GSE104165. The right half of the samples belong to the GBC group, and the left half of that belong to the normal group. GBC, gallbladder carcinoma; NGB, normal gallbladder; miRNAs, microRNAs; DE-miRNAs, differentially expressed miRNAs.

**Fig. 3:**
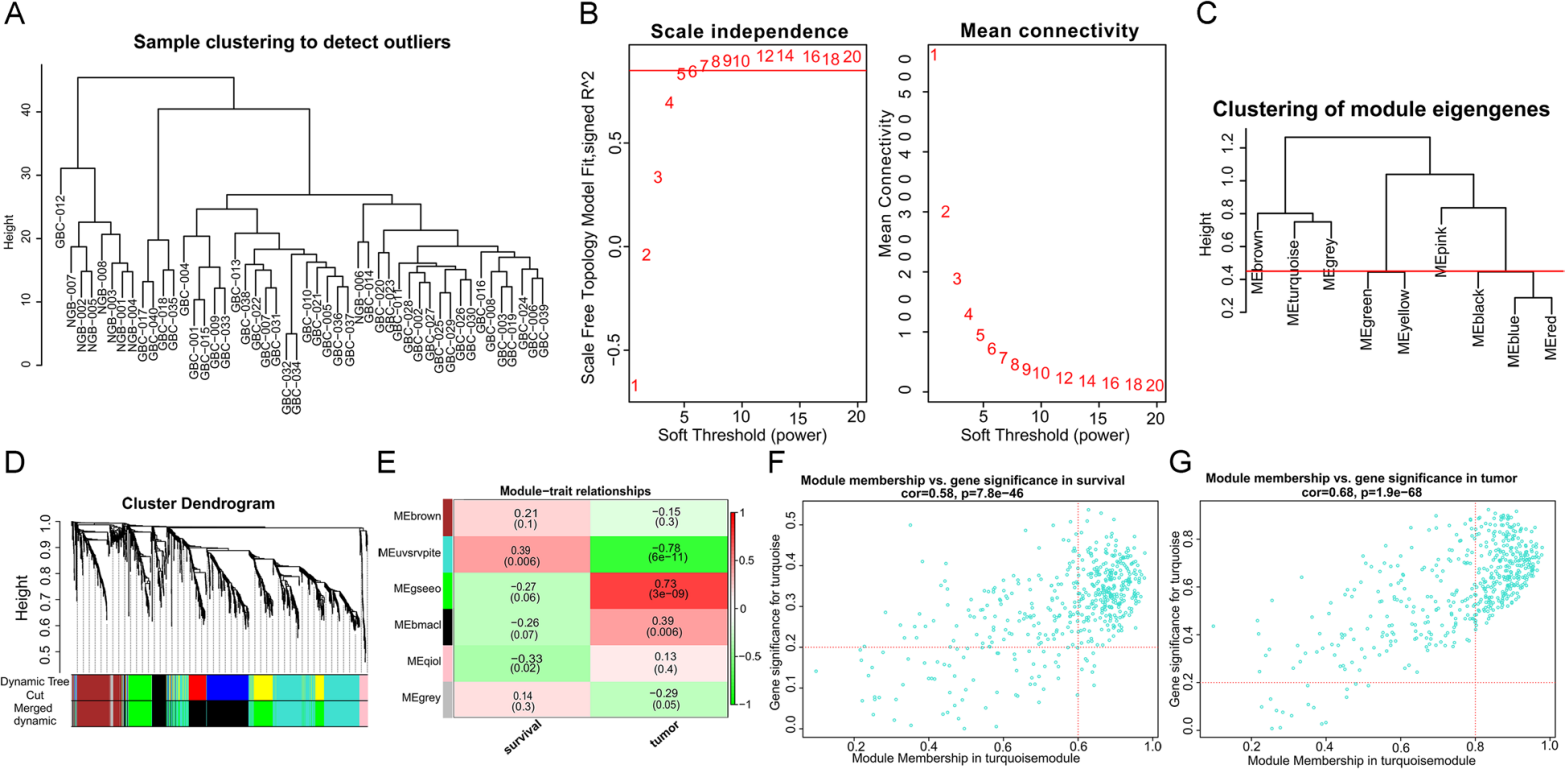
Construction of weighted co-expression network and mining hub genes. **A** Sample clustering. To identify and remove outliers. **B** Soft threshold selection process. 7.0 was selected as the optimal soft threshold with an R^2^=0.85. **C**, **D** Cluster dendrogram. Divided the miRNAs into different modules according to dynamic tree cutting algorithm; Each color represents one specific co-expression module; After merging, a total of six modules were generated. **E** Heatmap of the correlation between clinical traits (survival and tumor) and modules; Red represents positive correlation, green represents negative correlation, and the color shade represents the degree of correlation. **F**-**G** Hub miRNAs selection. The upper right quadrant is hub miRNAs; 175 miRNAs related to tumor and 169 miRNAs related to survival in turquoise module; The criteria were |MM|>0.8 and |GS|>0.2. miRNAs, microRNAs

**Fig. 4:**
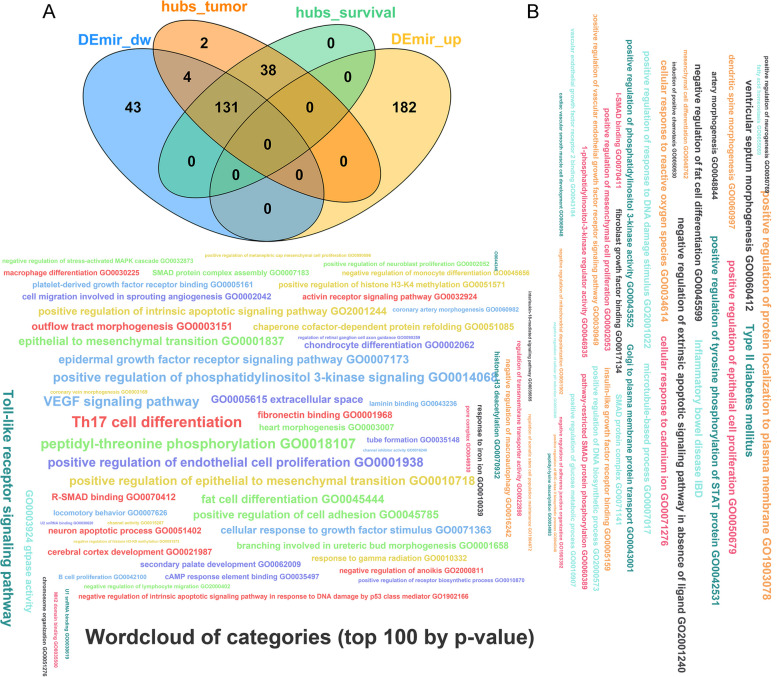
Exploration of 131 GBC-related DE-miRNAs. **A** VENN diagram to display the 131 GBC-related DE-miRNAs. **B** Wordcloud plot for these GBC-related DE-miRNAs. GBC, gallbladder carcinoma; DE-miRNAs, differentially expressed miRNAs

To authenticated the GBC-related DE-miRNAs, we conducted differential expression analysis firstly. Based on the miRNA sequencing data in the GSE104165 dataset, we detected 360 DE-miRNAs in GBC samples compared to NGB samples using the “limma” R package, including 182 up-regulated miRNAs and 178 down-regulated miRNAs (Fig. 2, Table S1). To further mine the miRNAs associated with GBC, we performed WGCNA using the data in the GSE104165 dataset. No obvious outliers were excluded by cluster analysis (Fig. 3A). 7.0 was selected as the optimal soft threshold with an R^2^ = 0.85 (Fig. 3B). Based on the optimal soft threshold, we divided the miRNAs into different modules according to dynamic tree cutting algorithm. After merging, a total of six modules were generated (Fig. 3C and D). Correlations between modules and clinical traits (survival and tumor) were calculated (Fig. 3E). The turquoise module was considered as the key module as the |correlation coefficient| between module and clinical traits (survival and tumor) was the highest (Fig. 3E). According to the criterion of |MM|>0.8 and |GS|>0.2, 175 miRNAs related to tumor in turquoise module, and 169 miRNAs related to survival in turquoise module were authenticated as hub miRNAs, namely GBC-related miRNAs (Fig. 3F and G). Hence, 131 GBC-related DE-miRNAs were obtained by taking the intersection of hub miRNAs related to tumor, hub miRNAs related to survival, up-regulated miRNAs, and down-regulated miRNAs (Fig. 4A, Table S2) The results from the miRNA enrichment analysis showed that those dysregulated miRNAs were mainly associated with VEGF signaling pathway and positive regulation of endothelial cell proliferation (Fig. 4B, Fig. S1 and Table S3), which were considered as important for GBC progression.

### Characteristic miRNAs for GBC

**Fig. 5:**
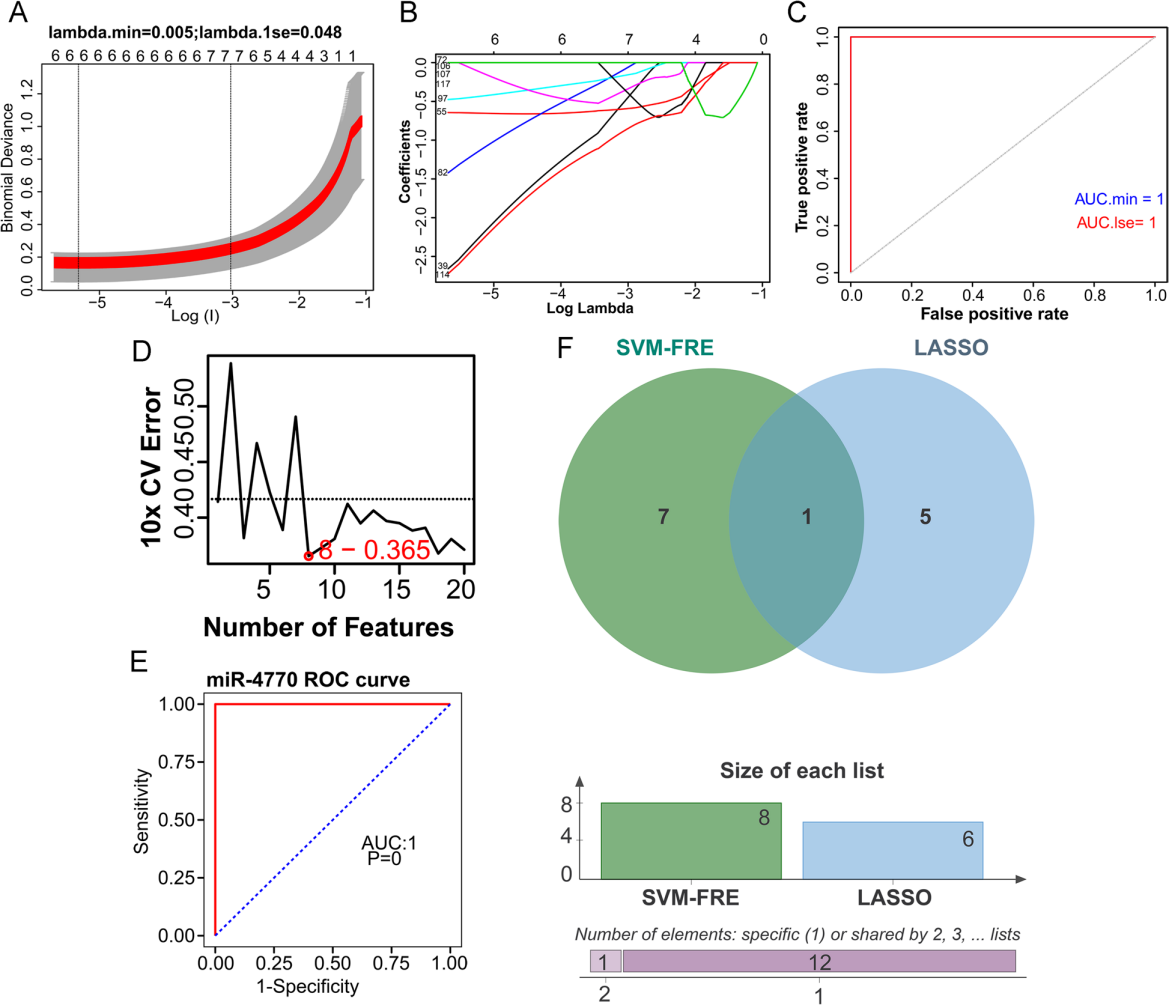
Identification of characteristic miRNAs for GBC based on machine learning. **A**, **B** LASSO algorithm. Using the LASSO algorithm, we identified six characteristic miRNAs. **C** ROC curve of LASSO model. To assess the diagnostic sensitivity and specificity of the LASSO model. **D** SVM-RFE algorithm. We identified eight characteristic miRNAs by the SVM-RFE algorithm. **E** Venn diagram of LASSO and SVM-RFE. To display the overlapped characteristic miRNA: hsa-miR-4770. **F** ROC curve of hsa-miR-4770. To investigate the diagnostic ability of hsa-miR-4770. miRNAs, microRNAs; GBC, gallbladder carcinoma; DE-miRNAs, differentially expressed miRNAs; LASSO, least absolute shrinkage and selection operator; SVM-RFE, support vector machine-recursive feature elimination; ROC, receiver operating characteristic

To determine the characteristic miRNAs from the 131 GBC-related DE-miRNAs, we adopted LASSO and SVM-RFE algorithms in GSE104165 dataset. Six characteristic miRNAs (hsa-miR-154-5p, hsa-miR-204-5p, hsa-miR-320b, hsa-miR-365a-3p, hsa-miR-379-5p, and hsa-miR-4770) were discerned using the LASSO algorithm (Fig. 5A and B). ROC curve with area under curve (AUC) value was 1.0 indicated the LASSO model performed well (Fig. 5C). Meanwhile, eight characteristic miRNAs (hsa-miR-5701, hsa-miR-125a-5p, hsa-miR-148b-3p, hsa-miR-128, hsa-miR-4770, hsa-miR-654-3p, hsa-miR-34a-5p, and hsa-miR-374a-5p) were selected with the SVM-RFE algorithm (Fig. 5D). Hence, one miRNA, namely hsa-miR-4770, was defined as characteristic miRNA for GBC by overlapping the miRNAs derived from these two algorithms (Fig. 5E). In order to investigate the diagnostic ability of the characteristic miRNA, we plotted the ROC curve. The AUC value of ROC curve was 1.0, indicating that the characteristic miRNA could distinguish GBC and NGB samples powerfully (Fig. 5F). The result indicated that hsa-miR-4770 had potential diagnostic value in clinical practice.

### The ceRNA network of characteristic miRNA

**Fig. 6:**
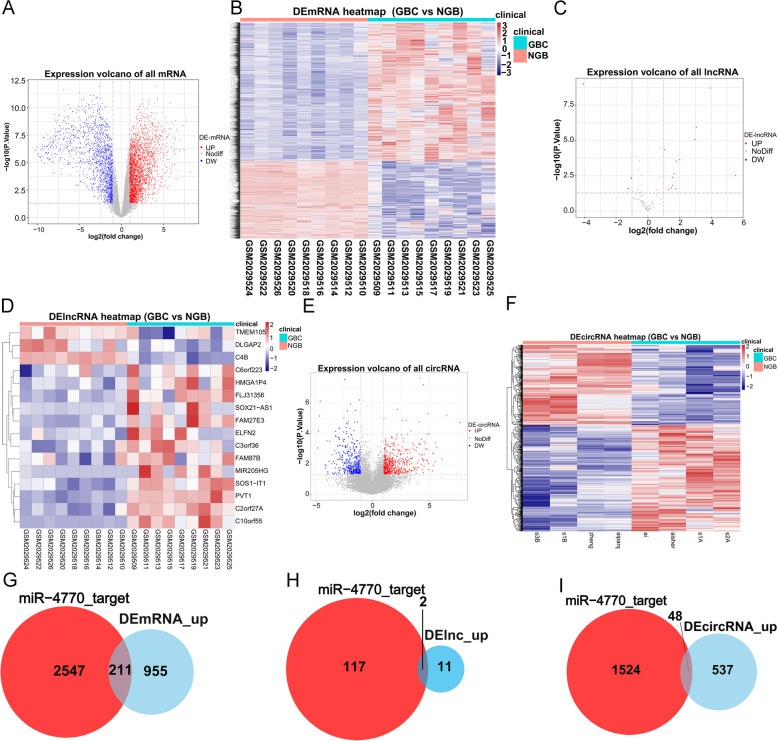
Identification of DE-mRNAs, DE-lncRNAs, and DE-circRNAs between GBC and NGB samples. **A**, **B** The volcano plot and heatmap exhibited the expression levels of 2758 up-regulated and 1547 down-regulated DE-mRNAs of each GBC sample in the GSE76633 dataset. **C, D** The volcano plot and heatmap from the GSE76633 dataset demonstrated 13 up-regulated and three down-regulated DE-lncRNAs in GBC samples. **E-F** The volcano plot and heatmap for 585 up-regulated and 443 down-regulated DE-circRNAs in the GBC samples from the GSE100363 dataset. **G** Venn diagram to show the overlapping of predicted targeting miRNA and up-regulated mRNAs. **H** Venn diagram to show the overlapping of targeting miRNA and up-regulated lncRNAs. **I** Venn diagram to show the overlapping of targeting miRNA and up-regulated circRNAs. DE-mRNAs, differentially expressed mRNAs; DE-lncRNAs, differentially expressed lncRNAs; DE-circRNAs, differentially expressed circRNAs; GBC, gallbladder carcinoma; NGB, normal gallbladder; DE-miRNAs, differentially expressed miRNAs; miRNA, microRNA; lncRNAs, long noncoding RNAs; circRNAs, circularRNAs.

**Fig. 7:**
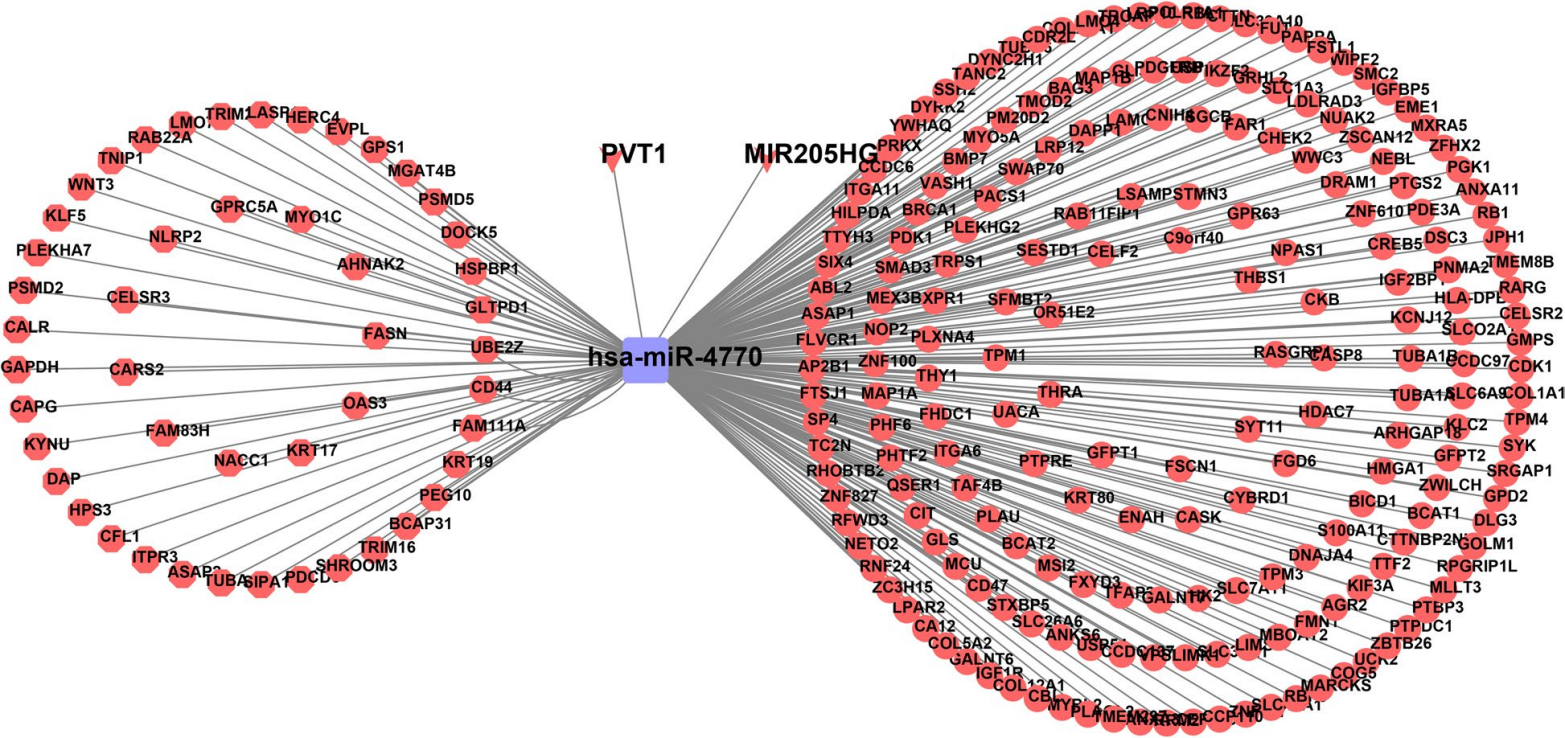
The construction of a ceRNA network including 211 mRNAs, one miRNA, 48 circRNAs and two lncRNAs. The square represents the hsa-miR-4770; The triangular diamond represents lncRNA; The dot represents the mRNAs; The hexagon represents the circRNA; Blue and red represent downregulation and downregulation. ceRNA, competing endogenous RNA; miRNA, microRNA; circRNAs, circularRNAs; lncRNAs, long noncoding RNAs

To explore the ceRNA regulation mechanisms of characteristic miRNA in GBC, we first screened for DE-mRNAs, DE-lncRNAs, and DE-circRNAs between GBC and NGB samples in GSE76633 and GSE100363 datasets. 2758 up-regulated mRNAs and 1547 down-regulated mRNAs in GBC were identified in the GSE76633 dataset (Fig. 6A and B). Meanwhile, 13 up-regulated lncRNAs and three down-regulated lncRNAs in GBC samples were mined (Fig. 6C and D). In the GSE100363 dataset, 585 up-regulated circRNAs and 443 down-regulated circRNAs in the GBC samples were determined (Fig. 6E and F). Using the Starbase database, we predicted 1166 mRNAs targeted by hsa-miR-4770, while 119 lncRNAs and 1572 circRNAs were predicted to target hsa-miR-4770. Since hsa-miR-4770 was down-regulated in the GBC group, we intersected the predicted targeting mRNAs, upstream lncRNAs, upstream circRNAs, and up-regulated mRNAs, lncRNAs, circRNAs respectively, to obtain a total of 211 mRNAs, two lncRNAs, and 48 circRNAs (Fig. 6G and I). Thus, a final ceRNA network containing 211 mRNAs, one miRNA, two lncRNAs, and 48 circRNAs was created (Fig. 7, Table S4).

**Fig. 8:**
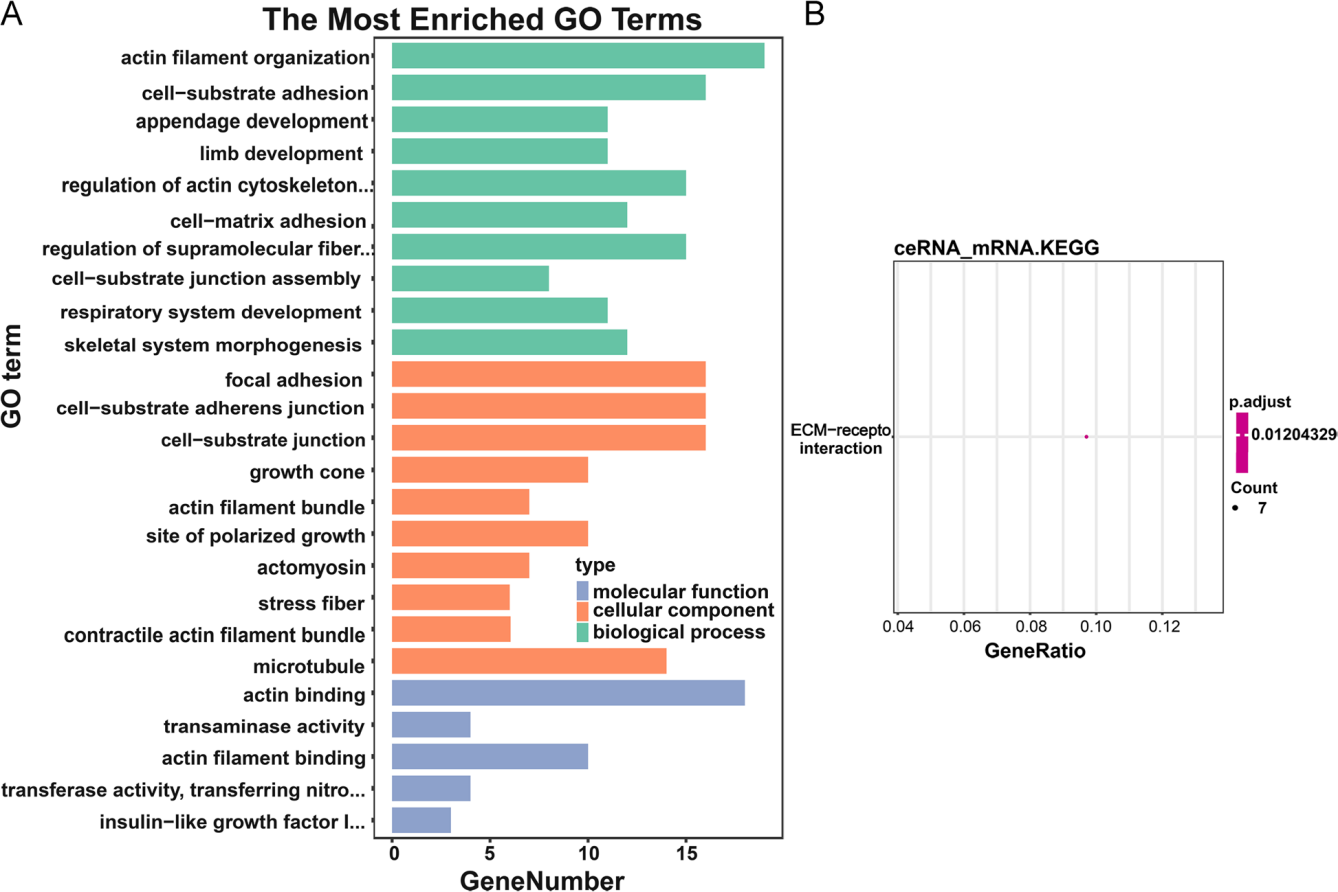
Functional enrichment analysis. **A** The histogram of enriched GO terms with statistical differences (*P*<0.05). **B **Bubble diagram of enriched KEGG pathway. GO, Gene Ontology; KEGG, Kyoto Encyclopaedia of Gene and Genome

To further understand the function and the pathways involved in targeting mRNAs in ceRNA regulatory network of the characteristic miRNA, we performed the corresponding functional enrichment analysis. A total of 80 biological process (BP) entries, 21 cellular components (CC) entries and five molecular functions (MF) entries, and one KEGG pathway were enriched (Tables S5, S6, S7, S8). The enriched GO terms with statistical differences (*P*<0.05) were listed (Fig. 8). We noted that the downstream genes regulated by hsa-miR-4770 were mainly involved in actin filament organization, cell-substrate adhesion, cell-matrix adhesion, regulation of reactive oxygen species metabolic process, glutamine metabolic process, and extracellular matrix (ECM) receptor interactions pathway-receptor interaction pathway.

### Key genes in the ceRNA network

**Fig. 9:**
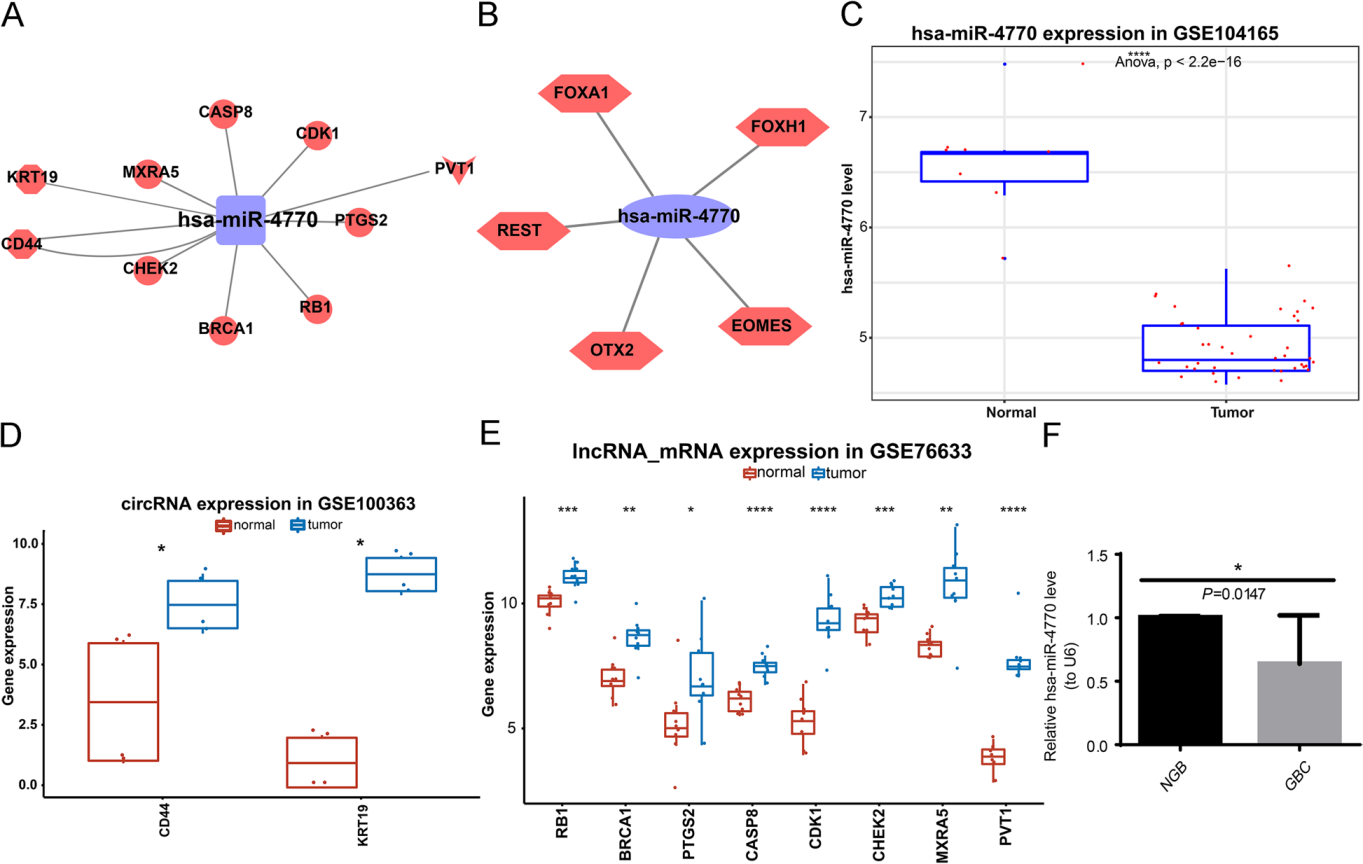
Investigation of hsa-miR-4770-related regulatory network. **A** The construction of ceRNA subnetwork based on top 10 key genes and hsa-miR-4770. The square represents hsa-miR-4770; The triangular diamond represents lncRNA; The dot represents the mRNAs. The hexagon represents the circRNA; Blue and red represent downregulation and downregulation. **B** The miRNA-transcription factor (TFs) network. The hexagons represent TFs, and the ellipse represents hsa-miR-4770. **C-E** The expression levels of 10 key genes and hsa-miR-4770 between GBC and NBG groups. **C** The hsa-miR-4770 was down-regulated in GBC; (**D**) CD44 and KRT19 were up-regulated in GBC; (**E**) RB1, BRCA1, PTGS2, CASP8, CDK1, CHEK2, MXRA5, and PVT1 were up-regulated in GBC. **F** The expression of hsa-miR-4770 at transcriptional level. The hsa-miR-4770 was reduced in GBC samples (*P*=0.0147). ceRNA, competing endogenous RNA; lncRNA GBC, long noncoding RNA; circRNA, circularRNA; TFs, transcription factors; gallbladder carcinoma; NGB, normal gallbladder

To explore the correlation between genes in the ceRNA network of hsa-miR-4770 and disease, we used varElect. Using the key words “gallbladder carcinoma” and “cancer”, we identified the top 10 genes, namely BRCA1, CHEK2, RB1, CASP8, PTGS2, CD44, KRT19, CDK1, PVT1, and MXRA5 were most correlated with the disease, in which PVT1 encoded lncRNA (Table 2). Therefore, we presented the ceRNA subnetwork composed of these 10 key genes and hsa-miR-4770 (Fig. 9A). In the network, PVT1 regulated the transcription of BRCA1, CHEK2, RB1, CASP8, PTGS2, CD44, CDK1, PVT1 and MXRA5 by targeting hsa-miR-4770. Besides, hsa-miR-4770-related TFs were further predicted according to the TransmiR database (Fig. 9B), suggesting the potential effects of EOMES, FOXA1, FOXH1, OTX2, REST on GBC by regulating hsa-miR-4770 (Table S9). In GBC, hsa-miR-4770 was down-regulated and the corresponding CD44, KRT19, RB1, BRCA1, PTGS2, CASP8, CDK1, CHEK2, MXRA5, and PVT1 were up-regulated (Fig. 9C and E). Then, we collected 10 NGB tissue samples and 10 GBC tissue samples from clinic for further validation of the expression of characteristic miRNA at transcriptional level. Consistent with the result of GSE104165 dataset, hsa-miR-4770 was significantly reduced in GBC samples compared with NGB samples (*P*=0.0147) (Fig. 9F).

**Table 2:** Top 10 genes selected from ceRNA network of characteristic miRNA by varElect

Symbol	Description	type	Matched phenotypes	Matched phenotypes count	Score	Log_10_(p)	Average Disease Causing Likelihood
BRCA1	BRCA1 DNA Repair Associated	Protein	cancer	1	144.19	4.11	43.0
CHEK2	Checkpoint Kinase 2	Protein	cancer	1	81.58	3.37	42.5
RB1	RB Transcriptional Corepressor 1	Protein	gallbladder carcinoma, cancer	2	71.86	2.85	67.0
CASP8	Caspase 8	Protein	cancer	1	39.55	2.71	68.6
PTGS2	Prostaglandin-Endoperoxide Synthase 2	Protein	gallbladder carcinoma, cancer	2	28.05	2.05	66.5
CD44	CD44 Molecule (Indian Blood Group)	Protein	gallbladder carcinoma, cancer	2	22.23	1.86	34.3
KRT19	Keratin 19	Protein	gallbladder carcinoma, cancer	2	21.08	1.83	48.7
CDK1	Cyclin Dependent Kinase 1	Protein	gallbladder carcinoma, cancer	2	21.05	1.83	79.7
PVT1	Pvt1 Oncogene	ncRNA	gallbladder carcinoma, cancer	2	18.06	1.74	ND
MXRA5	Matrix Remodeling Associated 5	Protein	cancer	1	17.8	1.94	3.8

**Fig. 10:**
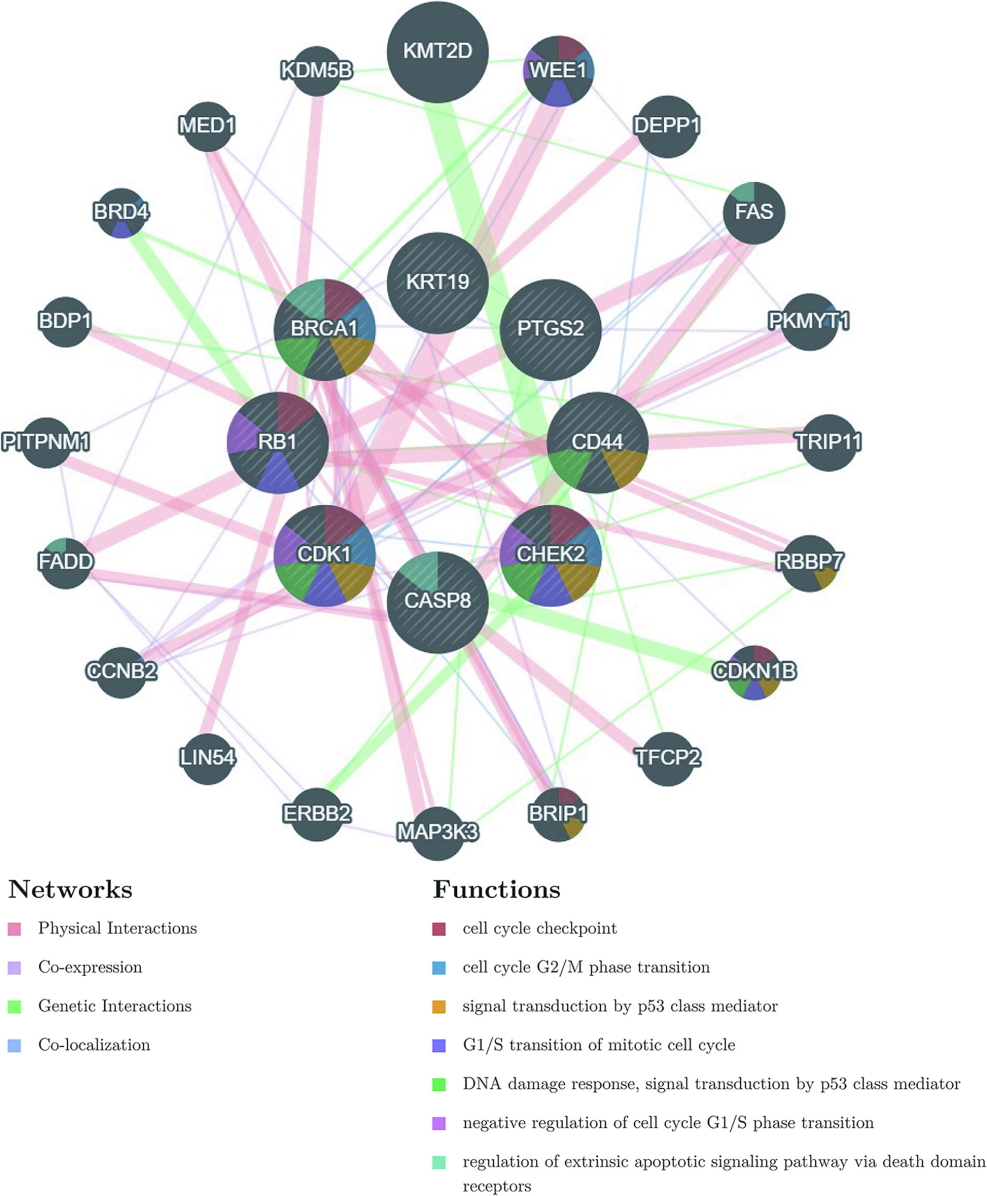
The PPI network developed by GeneMANIA. The purple border: Co-expression; The light red border: Physical Interactions; The light yellow: Prediction; The light green: Shared protein domains; The different sectors represent the functional pathways enriched by the gene. PPI, protein-protein interaction

In order to further investigate the interactions and functions of key genes in the ceRNA network of hsa-miR-4770, we performed a relevant analysis using the GeneMANIA database. The interactions of key genes and associated genes were shown. Among these genes, multiple genes were involved in cell cycle-related processes, p53 related pathway, and extrinsic apoptotic signaling pathway, which played an important role in the development of GBC (Fig. 10).

## Discussion

GBC originates from the gallbladder epithelial cells and stimulated by various factors such as gallbladder stones and chronic inflammation. GBC is the most common malignant tumor of hepato-biliary tract cancer, and has a high degree of malignancy, unsatisfactory survival, and prognosis [1]. Because of the absence of specific symptoms in the early stages of GBC, the vast majority of patients are diagnosed at advanced stages, and the five-year survival rate is only 10% [3]. Research on the diagnosis and treatment of GBC has never stopped, and miRNAs are receiving increasing attention. The miRNAs have been widely reported to play crucial roles in development, metastasis, epithelial-mesenchymal transition, and prognosis of GBC [9, 25–28].

Integrated bioinformatics approaches have helped to unravel the mechanisms and progression of cancer and have also facilitated research advances in cancer diagnosis, treatment and prognosis [29, 30]. In this study, we took the intersection of hub miRNAs identified by WGCNA and DE-miRNAs and obtained a total of 131 GBC-related DE-miRNAs. Those dysregulated miRNAs were mainly associated with VEGF signaling pathway and positive regulation of endothelial cell proliferation, which were considered as important for GBC progression. It has been shown that tumors can generate lymphatic vessels through the VEGF signaling pathway, thus affecting the distant metastasis of primary tumor cells [31]. Next, we overlapped the miRNAs derived from LASSO and SVM-RFE and defined hsa-miR-4770 as the characteristic miRNA for GBC. The ROC curve showed that hsa-miR-4770 has prominent diagnostic efficacy, which can clearly distinguish GBC samples from NGB samples, it also further illustrates that hsa-miR-4770 can be used as the diagnostic factor.

It has been reported that hsa-miR-4770 expression significantly changed in a wide variety of tumors. Shi-Han Xiao et al. found that hsa-miR-4770 was upregulated in tumor sediments of colorectal cancer. Based on six miRNAs including hsa-miR-4770, they constructed the signature nomogram for preoperative prediction of tumor deposits in colorectal cancer [32]. However, Xiao-Bing Wu et al. conducted a comparison of miRNAs between colorectal cancer at different stages and paired adjacent normal mucosa, and found that hsa-miR-4770 was significantly downregulated in stages II, II-III, and III [33]. Coincidentally, another study of colorectal adenocarcinoma also found significant downregulation of hsa-miR-4770 [34]. Yan-Yan Liu et al. identified that hsa-miR-4770 as one of the specific miRNAs in Helicobacter pylori (+) gastric cancer, showed a large number of interactions in the ceRNA network of Helicobacter pylori (+) gastric cancer, and downregulated significantly [35]. Fang-Yun Bai et al. showed that hsa-miR-4770 was downregulated in gastrointestinal stromal tumor cells by qRT-PCR [36]. Elena Vila Navarro et al. sequenced and validated the miRNAs used to detect pancreatic tumors and found that hsa-miR-4770 was significantly upregulated in pancreatic ductal adenocarcinoma, and identified hsa-miR-4770 as one of the novel markers with potential diagnostic or therapeutic value [37]. In addition, the downregulation of hsa-miR-4770 expression was also reported in esophageal squamous cell carcinoma, endometric cancer, and laryngeal squamous cell carcinoma [38–40]. We uncovered that hsa-miR-4770 was significantly downregulated in GBC compared with normal tissues in the GSE104165 dataset. We further performed qRT-PCR validation on hsa-miR-4770 expression, which yielded the same trend of variation. Hence, after a series of analyses and validations, we identified hsa-miR-4770 for the first time as a characteristic miRNA of GBC.

Through prediction and differential analysis, we constructed a ceRNA network of hsa-miR-4770 containing 211 mRNAs, one miRNA, two lncRNAs, and 48 circRNAs. GO and KEGG pathway analysis has been widely used to evaluate the enriched biological functions. GO enrichment of mRNAs regulated by hsa-miR-4770 showed its involvement in regulating actin filament organization, cell-substrate adhesion, cell-matrix adhesion, regulation of reactive oxygen species metabolic process, glutamine metabolic process. Furthermore, KEGG pathway enrichment analysis suggested that mRNAs regulated by hsa-miR-4770 was involved in the ECM receptor interactions pathway. ECM is known to play a crucial role in regulating cell survival, proliferation, migration, and differentiation [41]. These results revealed clues for the role of hsa-miR-4770 in GBC, and provided us with directions and ideas in subsequent studies.

We used GeneMANIA database to analyze the interaction and function of the above ceRNA sub-network, and found that hsa-miR-4770 target gene was involved in the cell cycle, DNA damage repair and p53 process. It is known that p53, as an oncogene, plays a key role in controlling cancer development and progression by regulating cell cycle arrest, apoptosis, senescence and DNA repair [42]. The p53 pathway maintains genome stability by regulating cell cycle delays and DNA damage repair [43]. It is thus clear that hsa-miR-4770 may participate in these biological processes by regulating its target genes, and then affect the occurrence and development of GBC, including the survival and prognosis of GBC patients.

We further constructed hsa-miR-4770 miRNA TFs network to predict the regulatory mechanism, and also paid attention to the research progress of TFs obtained. EOMES is mainly expressed in the nucleus of activated CD8^+^ T cells [44], which can enhance the proliferation and survival ability of tumor antigen-specific CD8^+^ T cells in immunotherapy, effectively control tumor growth or completely eliminate tumors [45]. FOXA1 and FOXH1 are members of FOX family of transcription factors, FOXA1 is decreased in hepatocellular carcinoma [46], FOXH1 promotes cell proliferation, invasion and tumorigenesis by enhancing the Wnt/β-catenin pathway in lung cancer cells and tissues [47]. OTX2 controls the regulatory landscape medulloblastoma through cooperative activity at enhancer elements and contributes to the expression of critical target genes [48]. REST has been found to be an important negative transcriptional regulator in various cells. In non-nerve cells and tumors (such as lung, breast and colon), REST plays an anti-cancer role [49], but its research in GBC has not been reported. The above TFs provides a little reference value for us to preliminarily reveal the regulation mechanism of hsa-miR-4770.

In the aforementioned results, we have only preliminarily explored the expression changes, ceRNA network of has-miR-4770 and interaction mechanism. Due to insufficient clinical samples and funding, we only included 10 pairs of samples for qRT-PCR detection and validation of the characterized miRNA. We will conduct more experimental validation of the mechanisms involved in the regulation of hub genes by miRNAs and TFs in the future to further explore and clarify their potential mechanisms.

## Conclusion

Taken together, we firstly identified hsa-miR-4770 as the characteristic miRNA for GBC based on the GEO database, and further constructed a ceRNA network for hsa-miR-4770. The regulatory pathways include cell cycle-related processes, p53, and extrinsic apoptotic signaling pathways. We preliminarily verified that hsa-miR-4770 expression was downregulated in GBC. Five key TFs involved in the regulation of hsa-miR-4770 were observed in the ceRNA network. This study provided some basis for understanding the underlying pathogenesis of GBC and identifying diagnostic biomarkers and potential therapeutic targets for GBC.

### Supplementary Information

**Additional file 1: Figure S1.** Heatmap plot for the enriched pathways by miEAA.

**Additional file 2: Table S1.** Total differentially expressed miRNAs in GBC and normal tissues.

**Additional file 3: Table S2.** The 131 GBC-related DE-miRNAs.

**Additional file 4: Table S3.**The miRNA enrichment analysis results of dysregulated miRNAs.

**Additional file 5: Table S4.** The ceRNA network of GBC containing 211 mRNAs, 1 miRNA, 2 lncRNAs, and 48 circRNAs.

**Additional file 6: Table S5.** Eighty  biological process entries analysis of ceRNA regulatory network.

**Additional file 7: Table S6.** Twenty-one cellular components analysis of ceRNA regulatory network.

**Additional file 8: Table S7.** Five molecular functions entries analysis of ceRNA regulatory network.

**Additional file 9: Table S8.** One KEGG pathway analysis of ceRNA regulatory network.

**Additional file 10: Table S9.** The potential effects of EOMES, FOXA1, FOXH1, OTX2, REST on GBC by regulating hsa-miR-4770.

## Data Availability

Data analyzed in this study is publicly available from GEO database (Accession No.: GSE104165, GSE76633 and GSE100363).

## Abbreviations

GBC: Gallbladder carcinoma
ceRNA: Competing endogenous RNA
DE-miRNAs: Differentially expressed miRNAs
WGCNA: Weighted gene co-expression network analysis
LASSO: Least absolute shrinkage and selection operator
SVM-RFE: Support vector machine-recursive feature elimination
circRNAs: Circular RNAs
lncRNAs: Ng noncoding RNAs
GO: Gene Ontology
qRT-PCR: Quantitative real-time polymerase chain reaction
ncRNAs: Noncoding RNAs
GEO: Gene Expression Omnibus
MREs: miRNA response elements
NGB: Normal gallbladder
DE-mRNAs: Differentially expressed mRNAs
DE-lncRNAs: Differentially expressed lncRNAs
DE-circRNAs: Differentially expressed circRNAs
ROC: Receiver operating characteristic
BP: Biological processes
CC: Cellular components
MF: Molecular functions
TFs: Transcription factors
