# Supplementary material for: Identification of characteristic genes and construction of regulatory network in gallbladder carcinoma
Source: BMC Med Genomics. 2023 Oct 11;16:240. doi: 10.1186/s12920-023-01663-z (PMC10566037; doi:10.1186/s12920-023-01663-z)

Figure S1. Heatmap plot for the enriched pathways by miEAA.

The positive regulation of endothelial cell proliferation, which were considered as important for GBC progression. miEAA, miRNA Enrichment Analysis and Annotation Tool; GBC, gallbladder carcinoma.

miRNA / precursor to category heatmap (top 100 by p-value)

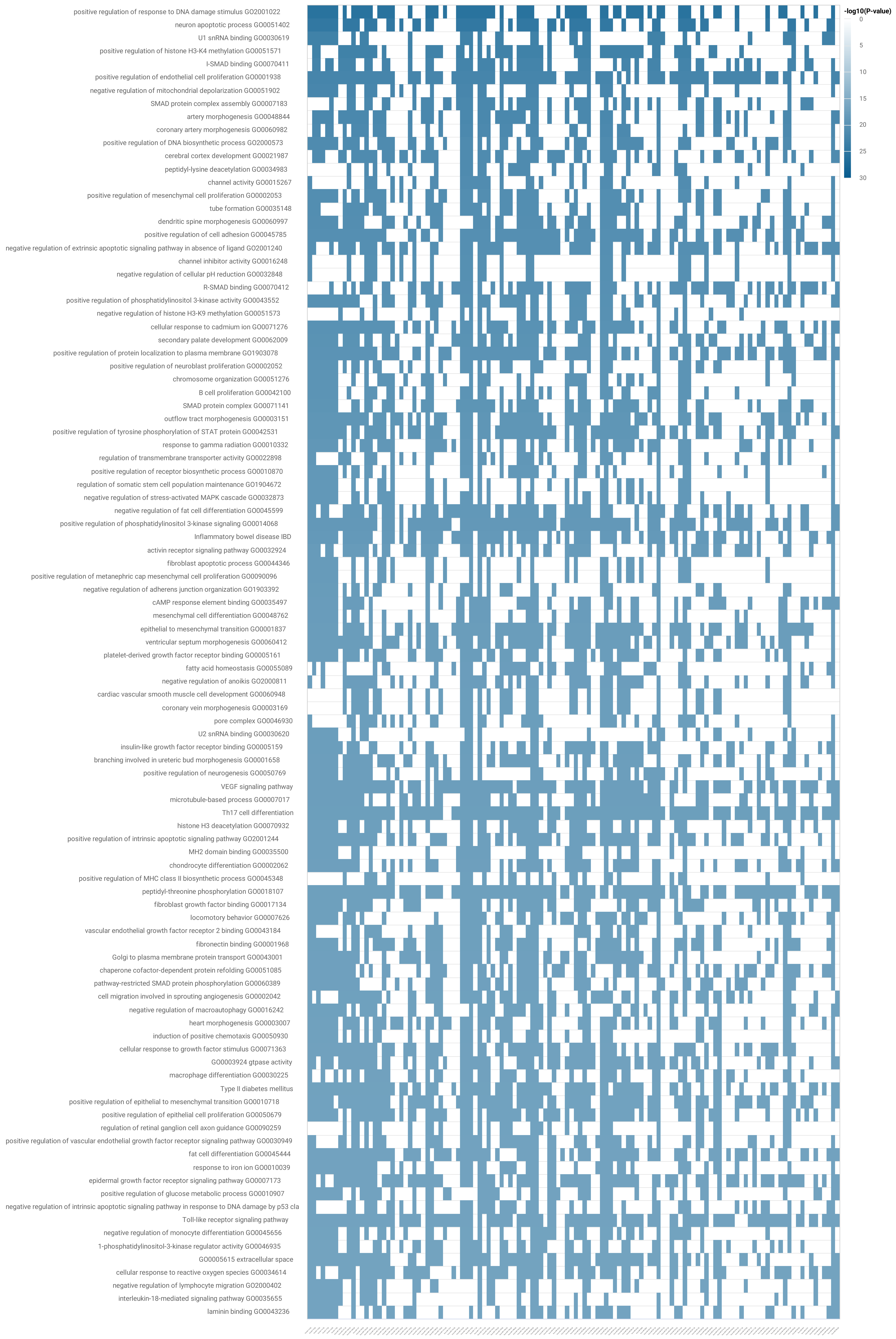

Supplement: Supplementary file 1 — Additional file 1: Figure S1. Heatmap plot for the enriched pathways by miEAA. [file 12920_2023_1663_MOESM1_ESM.pdf]
